# A Pressure Swing Approach to Selective CO_2_ Sequestration Using Functionalized Hypercrosslinked Polymers

**DOI:** 10.3390/ma14071605

**Published:** 2021-03-25

**Authors:** Alex M. James, Jake Reynolds, Daniel G. Reed, Peter Styring, Robert Dawson

**Affiliations:** 1 Department of Chemistry, University of Sheffield, Brook Hill, Sheffield S3 7HF, UK; alex.james@liverpool.ac.uk (A.M.J.); jreynolds5@sheffield.ac.uk (J.R.); 2 Department of Chemical and Biological Engineering, University of Sheffield, Mappin Street, Sheffield S1 3DJ, UK; Cpa08dr@sheffield.ac.uk (D.G.R.); p.styring@sheffield.ac.uk (P.S.)

**Keywords:** porous polymers, porous materials, carbons capture

## Abstract

Functionalized hypercrosslinked polymers (HCPs) with surface areas between 213 and 1124 m^2^/g based on a range of monomers containing different chemical moieties were evaluated for CO_2_ capture using a pressure swing adsorption (PSA) methodology under humid conditions and elevated temperatures. The networks demonstrated rapid CO_2_ uptake reaching maximum uptakes in under 60 s. The most promising networks demonstrating the best selectivity and highest uptakes were applied to a pressure swing setup using simulated flue gas streams. The carbazole, triphenylmethanol and triphenylamine networks were found to be capable of converting a dilute CO_2_ stream (>20%) into a concentrated stream (>85%) after only two pressure swing cycles from 20 bar (adsorption) to 1 bar (desorption). This work demonstrates the ease with which readily synthesized functional porous materials can be successfully applied to a pressure swing methodology and used to separate CO_2_ from N_2_ from industrially applicable simulated gas streams under more realistic conditions.

## 1. Introduction

The 2015 Paris Agreement aims to limit the average global temperature increase to 2 °C. One of the key causes of climate change is anthropogenic carbon dioxide, and recently, the UK government committed to a net zero emissions target by 2050. In the long term, the most effective method to lower carbon dioxide (CO_2_) emissions is to switch to renewable energy sources. However, the transition to renewable energy such as solar and wind is likely to take decades, hence the continued reliance on non-sustainable energy sources [1,2]. In order to meet the short- to medium-term emissions targets, the capture, storage and utilization of CO_2_ from large anthropogenic point sources such as fossil fuel power plants and the steel industry is key to mitigating the ever-increasing levels of CO_2_ in the atmosphere, thereby preventing irreversible climate change [2,3,4].

One of the key challenges facing materials for carbon capture from anthropogenic point sources is the low concentration of CO_2_ in flue gas streams—often around or lower than 20%. The remaining volume comprises largely N_2_ with smaller amounts of water vapor, oxygen, SO_2_ and NO_x_ (among others) [5,6,7,8]. In order to capture CO_2_ efficiently, any capture process therefore needs to show high selectivity toward CO_2_.

The current state-of-the-art industrial method of capturing CO_2_, dubbed amine scrubbing, has remained unchanged for decades and involves the use of aqueous solutions of amines such as monoethanolamine (MEA) [9]. This process relies on chemisorption, by which the MEA selectively reacts with CO_2_ to form a carbamate salt.

Over the past few years, there has been a move away from the amine scrubbing process due to significant and numerous drawbacks. These include the chemisorption process requiring very high temperatures (ca. 130 °C) to liberate the CO_2_ and regenerate the free amine. Attaining these high temperatures is a challenge for the industry and comes at a high price both fiscally and environmentally. Nonsensically, in order to power this process, one has to produce CO_2_ to capture CO_2_ [10]. Other issues include the corrosive nature of the amine solution, along with the sensitivity of such solvents to other gaseous impurities present in the flue gas, such as SO_x_ and NO_x_ [11,12,13]. This results in continuous degradation as well as evaporation, meaning the amine solution needs to be changed on a regular basis, thereby raising the operating cost of the process [14]. Due to the difference in the temperature at which the reaction of amines and CO_2_ react compared to the temperature required to regenerate the amine, this process is known as a temperature swing approach. However, the high energy penalty required by the regeneration temperature means this method is not ideal for the capture of CO_2_. In contrast, a physisorption process, whereby the interaction between adsorbent and adsorbate is weaker yet still significant enough for the binding of CO_2_ to the substrate surface, requires much less energy to regenerate the free material and liberate the pure gas [3,12].

Most reports of new materials for carbon capture use a temperature swing approach. There is much less literature relating to adsorbents using the alternative pressure swing approach. Pressure swing adsorption (PSA) technology is a growing body of research which is compatible with solid sorbents and has the potential to optimize and replace the current temperature swing technologies applied in industry [15,16]. In a pressure swing approach, CO_2_ is adsorbed at high pressures by a solid sorbent before being desorbed at lower pressures or under slight vacuum (VPSA). Different sorbents require different pressure profiles but are typically around 10–30 bar in the adsorption cycle. In comparison to temperature swing, PSA is an inherently low-energy technique for which high temperatures are not required during adsorption or desorption. PSA is also a much faster technique compared to temperature swing, as there is no thermal lag, meaning that the adsorb/desorb cycle can be performed rapidly. There is much scope for variation with PSA, such as optimization of the sorbent, the working pressures and temperatures of the process, all of which can be varied to yield the most efficient and effective system.

In order for a material to be considered a viable choice as a solid sorbent for pressure swing adsorption, certain criteria have to be met. These include the material being stable and selective toward CO_2_ at both low and high pressures. The material must demonstrate good recyclability over many pressure swing cycles. Furthermore, it is desirable for it to be both cheap and relatively simple to make with good yields due to the scale of the process and to keep the cost low.

Over the last two decades, as interest in carbon dioxide capture/utilization (CCS/CCU) has accelerated, numerous sorbents demonstrating CO_2_ capturing capabilities have been reported [17,18,19], mainly using the temperature swing approach [3,11,12,20]. These include zeolites [21], hybrid materials such as metal organic frameworks (MOFs) [22], activated carbons, ionic liquids [23,24] and microporous organic polymers (MOPs) [25,26,27,28,29,30,31].

MOPs are a family of porous materials composed solely of the lighter elements of the periodic table. There are a large number of different subclasses of MOP, such as conjugated microporous polymers (CMPs) [32,33,34], covalent organic frameworks (COFs) [35,36,37,38], covalent triazine frameworks (CTFs) [39,40,41,42,43] and polymers of intrinsic microporosity (PIMs) [44,45,46,47] which have been applied to various applications ranging from chemosensing [39,48,49,50] to catalysis [51,52,53,54] and waste water treatment [55,56,57,58]. CO_2_ uptakes of MOPs are typically measured at conditions of around 1 bar and at temperatures ranging from 273 to 298 K. Some of the best-performing MOPs include functionalized networks containing amine groups with uptakes of around 15–20 wt.% at 1 bar and 273 K [59,60]. At higher pressures, materials such as PAF-1 and PPN-4 have a reported uptake of 130 wt.% (40 bar, 298 K) [61] and 212 wt.% (50 bar, 295 K) [62], respectively. However, one class of MOP stands out for the application of carbon dioxide capture due to their low skeletal density, chemical and thermal stability and synthesis using cheap, readily available starting materials on a large scale—hypercrosslinked polymers (HCPs) [27,28,29,63]. At high pressures, there are, however, relatively few studies. HCPs based on 4,4′-bis(chloromethyl)-1,1′-biphenyl (BCMBP) were shown to have uptakes of up to 58.7 wt.% at 30 bar [28]. While this falls short of the PAF/PPN materials, HCP synthesis is considerably less complex, cheaper and more readily scalable.

Hypercrosslinked polymers are rigid porous networks with typical surface areas in the range of 500–2000 m^2^/g [64,65,66]. Their synthesis is often based on Friedel–Crafts chemistry using a Lewis acid catalyst such as iron (iii) chloride to yield a highly crosslinked and permanently microporous insoluble solid product. HCP synthesis requires the use of crosslinking groups, such as methyl chlorides, often dubbed “internal crosslinkers” [67,68], or external crosslinkers such as formaldehyde dimethyl acetal (FDA) [69]. This external “knitting method” allows potentially any rigid aromatic monomer to be hypercrosslinked.

Crucially, the “knitting method” provides a route for the incorporation of a range of chemical functionalities into the networks by polymerization of pre-functionalized monomers. This has led to the investigation of HCPs for a variety of different applications [27,70,71,72]. For CO_2_ capture, it is well known that different chemical moieties can impart increased selectivity toward CO_2_ over other gases due to more favorable interactions with the chemical moiety and the dipole of the CO_2_ [31,73,74,75]. These interactions are crucial to maximizing their selectivity toward CO_2_.

In this work we report the synthesis, characterization and implementation of functional HCP networks for use as solid sorbents using a PSA approach. The CO_2_ uptake capacity and uptake kinetics are measured at high pressure followed by measurements using simulated flue gas compositions. The CO_2_ selectivity of the materials is calculated, and the recyclability potential of the HCPs is evaluated. Further to this, in order to keep the study industrially applicable, all samples were exposed to more industrially relevant gas streams, and the materials themselves were exposed to the humid laboratory conditions and not merely used immediately after drying.

## 2. Results and Discussion

**Figure 1:**
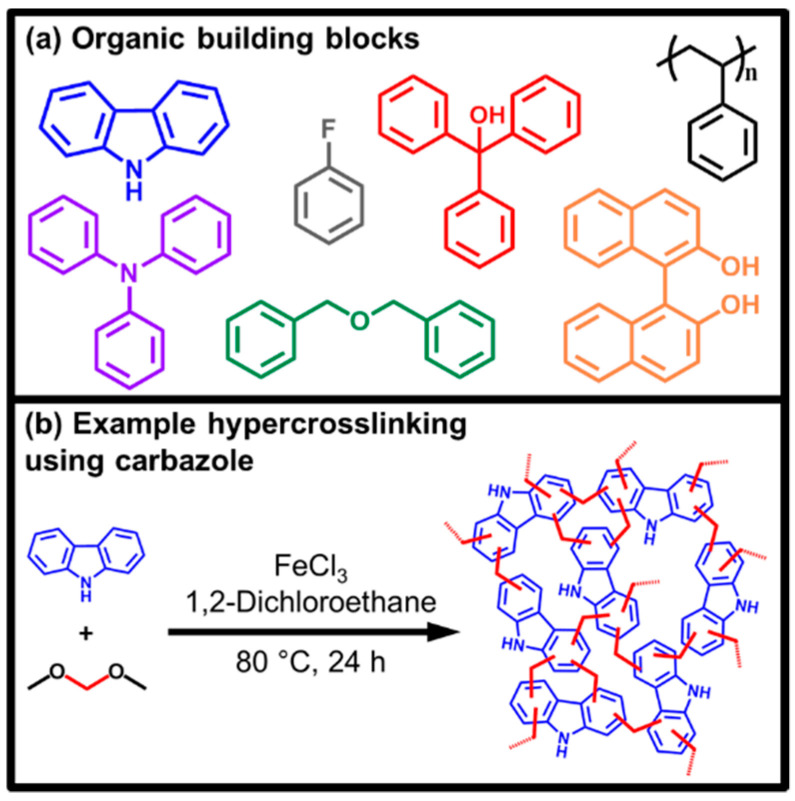
Schematic representation of hypercrosslinked polymer (HCP) synthesis using the so-called external crosslinking or “knitting” method. (**a**) Example monomers used in this work, poly(styrene), triphenylmethanol, BINOL, carbazole, triphenylamine, dibenzyl ether and fluorobenzene and (**b**) an example of hypercrosslinking synthesis.

Seven hypercrosslinked polymers were synthesized from functionalized monomers, all possessing different chemical moieties to study how these groups affected the CO_2_ uptake and selectivity at high pressure. Monomers including alcohol functionalities (triphenylmethanol and 1,1′-bi-2-naphthol (BINOL)) and amine functionality (2° amine carbazole and 3° amine triphenylamine), which have been previously shown to increase CO_2_ uptakes in porous polymers [24], were investigated. Other functionalities such as halogens (fluorobenzene) and a newly synthesized network based on dibenzyl ether were also investigated (Figure 1). Further to this, a non-functionalized network was synthesized from polystyrene which provides a non-functionalized network, which could also be synthesized from waste polymers [76]. This network provides a control with which to compare the functionalized networks. While hypercrosslinked polymers made from poly(styrene) [77], carbazole [78], BINOL [27], triphenylamine [79] and fluorobenzene [70] have previously been reported, this is, to our knowledge, the first reported synthesis of networks synthesized from dibenzyl ether and triphenylmethanol.

**Figure 2:**
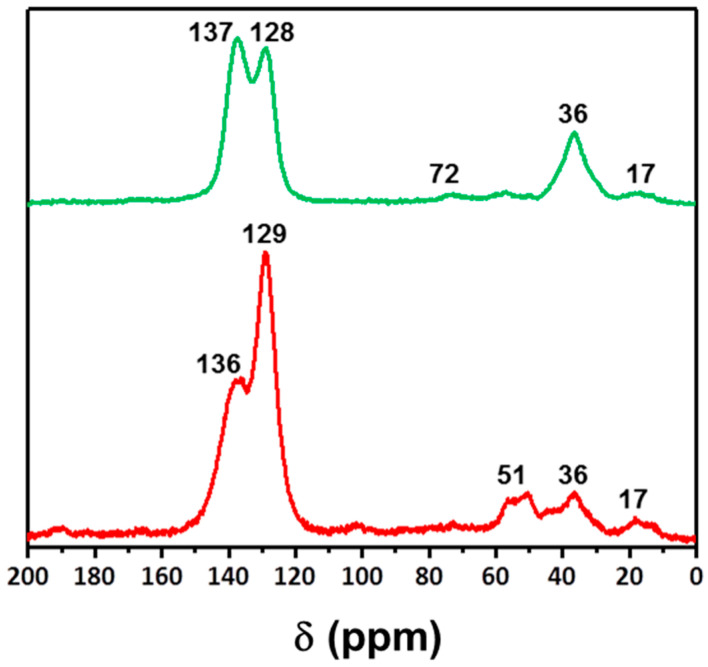
CP/MAS solid state ^13^C NMR spectra of the dibenzyl ether (above) and triphenylmethanol (below) networks.

All networks were obtained in good yields (Table S1) similar to previous reports of the same networks [27,69]. Structural characterization of the HCPs was performed by elemental analysis (Table S2), infrared spectroscopy (FT-IR) (Figure S1) and ^13^C solid state cross polarization magic-angle spinning (CP/MAS) NMR spectroscopy (ssNMR) (Figure 2 and Figure S2). Calculated %C, H and N of the networks were found to be typical for HCPs synthesized via Friedel–Crafts alkylation. There is some variation from the expected values as these are calculated assuming an idealized structure in which all protons have been exchanged for a methylene bridge. The presence of end groups and adsorbed molecules such as CO_2_ and water vapor may also contribute to the deviation from theoretical values. Furthermore, the presence of residual iron known to persist even after extensive purification procedures may also lead to errors in these values. However, incorporation of heteroatoms such as nitrogen indicates successful polymerization, and nitrogen values of 5.17% and 4.35% were observed for the carbazole and triphenylamine networks, respectively, indicating successful incorporation of amines into the structure.

Analysis by FTIR (Figure S1) suggests that the incorporation of the monomers into the networks was successful with characteristic signals at ca. 2800 cm^−1^ and 1600 cm^−1^ corresponding to the C–H and C=C stretches for each network. Additional signals at ca. 3500 cm^−1^ and ca. 1000 cm^−1^ are assigned to the –OH and ether stretch of the triphenylmethanol and dibenzyl ether network, respectively. ^13^C ssNMR spectra were collected for all samples and can be seen in Figure S2, while the spectra for the two newly synthesized materials are presented in Figure 2. All networks showed two prominent signals at ca. 140 and 130 ppm corresponding to quaternary aromatic carbons (C_Ar_) and aromatic C_Ar–H_. Signals at 36 ppm are assigned to methylene bridges in the networks. The resonance at 51 ppm for the triphenylmethanol network is assigned to the C–OH. For the dibenzyl ether network, a resonance at 72 ppm is assigned to the CH_2_-O–CH_2_ carbons adjacent to the ether linkage. A further resonance is observed at ca. 17 ppm and is attributed to unreacted end groups arising from incomplete reaction of the FDA crosslinker.

**Figure 3:**
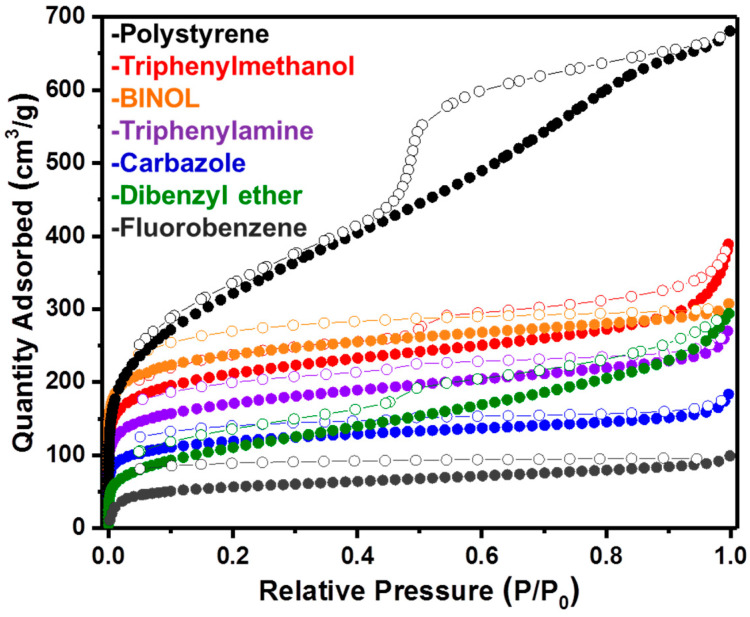
Full gas sorption isotherms for all polymer networks synthesized. Poly(styrene), triphenylmethanol, BINOL, carbazole, triphenylamine, dibenzyl ether and fluorobenzene.

The porosity of the networks was measured using nitrogen adsorption/desorption isotherms at 77 K (Figure 3). Brunauer-Emmett-Teller (BET) surface areas were calculated over a relative pressure range (P/P_0_) of 0.01–0.15 with the total pore and micropore volumes calculated at 0.95 and 0.1 P/P_0_, respectively (Table 1). All networks adsorbed large volumes of nitrogen at low relative pressure (<0.1 P/P_0_), indicating the presence of micropores. All networks demonstrated further uptake at higher partial pressures. This was particularly noticeable for the poly(styrene) network, which demonstrates a type II hysteresis loop on the desorb indicative of further larger (meso)pores, consistent with previously reports [77].

All samples were found to be porous with specific BET surface areas ranging from 213 m^2^/g to 1124 m^2^/g. The highest surface area was found to be derived from the polystyrene network and is similar to that reported previously in the literature [77]. Overall, the inclusion of functionality into the networks resulted in a lower surface area than non-functionalized HCPs. This is due to the functional groups sitting in the pores of the material and also the fact that different chemical moieties can hinder the efficacy of the hypercrosslinking process, thereby resulting in a less crosslinked network. Functional 3D monomers, however, such as BINOL, are still able to produce relatively high surface area networks. Despite their lower surface areas, the effects of the functionality are still interesting for CO_2_ capture and the potential for increased selectivity over nitrogen.

The total pore volumes of the materials ranged from 0.14 cm^3^/g to 1.01 cm^3^/g, with the fluorobenzene- and polystyrene-derived HCPs showing the lowest and highest pore volumes, respectively, as might be expected from the highest and lowest surface area networks. This is also consistent with the pore size distribution plots (Figure S3), which show that only micropores are present in the fluorobenzene network yet an abundance of mespores are micropores are present in the poly(styrene) network. As a proportion of pore volume (V_0.1_/V_tot_), both carbazole and fluorobenzene showed the largest contribution of micropores, while dibenzyl ether was found to have a larger proportion of meso- and macropores. It has been previously reported that smaller pores are preferential over larger pores for CO_2_ capture, particularly at lower pressures where the uptake has not reached a maximum. It was hypothesized that the networks with a larger % of micropores may therefore be better suited toward CO_2_ capture than those possessing larger pores at 25 bar [28].

### 2.1. Kinetic Uptake of CO_2_

High-pressure CO_2_ adsorption experiments were conducted using the setup as previously reported by Reed and co-workers [23,80]. Briefly, an adsorbent was packed into a sealed tube, which was exposed to high pressures of gas before being weighed to gravimetrically determine the CO_2_ uptake. All measurements were repeated three times and an average of the data was taken and used. All measurements on the functionalized HCPs were recorded at 40 °C to more closely match cooled flue gas temperatures from industrial sources. Stack temperatures can vary depending on the process but can range from 120 °C for post-combustion processes to 250–350 °C for steel plants and over 1000 °C for smelting works. As such, flue gas temperatures need to be reduced to values where absorption or adsorption are feasible. Moisture vapor is also an important consideration when for post-combustion capture [5,26]. All samples were therefore tested under “wet” conditions. More specifically, after synthesis, the samples were dried under vacuum at 60 °C to remove solvents before then being allowed to adsorb moisture from the air at 40–50% humidity for at least 24 h before any adsorption measurements were carried out. These conditions allow for results more comparable to those used in industry where gas mixtures are typically hydrated.

**Figure 4:**
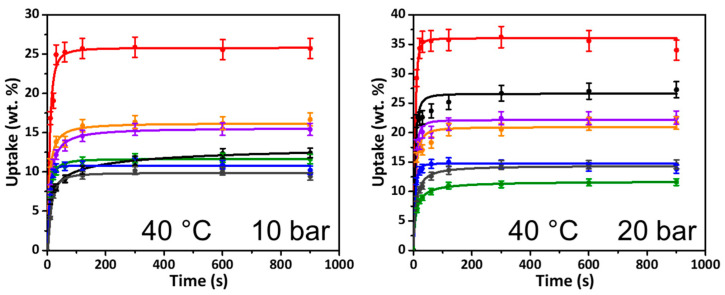
Kinetic studies of CO_2_ uptake, for poly(styrene), triphenylmethanol, BINOL, carbazole, triphenylamine, dibenzyl ether and fluorobenzene networks at 40 °C and 10 bar (**left**) and 20 bar (**right**).

Pressures of 10 and 20 bar are typical pressures for PSA, which are easily attainable without a significant increase in plant operating costs. The rate at which each network reached saturation at 10 and 20 bar was therefore measured (Figure 4). At 20 bar, all HCP networks become fully saturated rapidly with t_90_ values (the time at which 90% of the total uptake is completed) of 85 s or less (Table S5), while at the lower pressure of 10 bar, the time to reach saturation was up to 3 min, with the hydrophilic networks triphenylmethanol and BINOL taking longest and the hydrophobic networks poly(styrene) and fluorobenzene the shortest (Figure 4). The rapid sorption period is advantageous should these materials be applied to an industrial PSA approach given that the less time the material has to spend at elevated pressures, the greater the economic and energy benefit.

At 10 bar, the two –OH-containing networks (triphenylmethanol and BINOL) showed the highest final uptakes. Alcohol-containing porous polymers have previously been shown to demonstrate good CO_2_ capture capabilities, and these results further corroborate the advantage of such functionalities even at higher pressures [27,81]. This is closely followed by the triphenylamine network, which demonstrates uptake of close to 15% wt. The highest surface area material—the non-functional poly(styrene)—shows uptake at 10 bar at around 11% wt. This material has a much higher surface area than the two alcohol materials yet underperforms in comparison to these two networks. Finally, the carbazole, fluorobenzene and the newly synthesized dibenzyl ether networks all demonstrate less prominent uptakes ranging from 6% wt. to 10% wt.

At 20 bar, all samples show increased uptake of CO_2_ compared to 10 bar, which is to be expected. The triphenylmethanol network continues to show the highest final uptake, being close to 35% wt. However, the largest improvement in uptake is exhibited by the poly(styrene) network, with an uptake of just over 25% wt. This result perhaps highlights that at elevated pressures, it is the surface area which becomes more important than chemical functionality. The BINOL network shows a final uptake of just under 20% wt., as does the triphenylamine network. The newly synthesized dibenzyl ether network shows the poorest uptake at ~10% wt., which is not much of an improvement from the uptake reported at 10 bar. This poor performance, despite a reasonable surface area, could be somewhat due to the presence of larger pore sizes dominating the material. In comparison, the triphenylmethanol, carbazole and fluorobenzene networks have a greater proportion of smaller micropores aiding their uptake under these conditions. Finally, the carbazole and fluorobenzene networks both have uptakes of ~15% wt., which is a slight increase from the values recorded at 10 bar.

### 2.2. Selectivity Measurements

In order to investigate how selective networks were for CO_2_ over that of the major component of flue gas (N_2_), individual uptakes of both CO_2_ and N_2_ were measured for each HCP network at pressures between 5 and 25 bar at a temperature of 40 °C (Figures S4 and S5). HCP networks were exposed to a pressurized stream of either pure CO_2_ or N_2_ for a 5 min adsorption period, the time at which the previous kinetic runs were shown to be sufficient for equilibration, after which the gravimetric uptake was recorded, and the average uptake was calculated over three runs. Using these experiments, it is possible to estimate the CO_2_:N_2_ selectivity of the networks at high pressures typical for PSA.

All the networks demonstrated much higher uptakes of CO_2_ than N_2_ under identical adsorption conditions (Table 2), typically thereby demonstrating a preference toward adsorbing CO_2_ over N_2_. The nitrogen uptake of the networks correlates well with the BET surface areas of the materials, exhibiting no strong interaction with the network surface functionalities. By dividing the CO_2_ uptake by the N_2_ uptake, crude selectivity ratios can be calculated for each material, which serve as a rough guide as to the relative affinity the networks have for each gas. Perhaps unsurprisingly, the selectivity of each material decreases as pressure increases. As an example, the triphenylamine network has a selectivity of 9:1 at 10 bar, which decreases to 7:1 at 20 bar. Likewise, this is also true for the triphenylmethanol network, which has a calculated selectivity of 7:1 at 10 bar and 5:1 at 20 bar. The poly(styene) network, non-functional in nature, has the best selectivity of 13:1 and 9:1 at 10 bar and 0 bar, respectively. This selectivity is clearly not a result of chemical functionality and is most likely the result of the high surface area of the material and small pores, which preferentially adsorb CO_2_ over N_2_. The dibenzyl ether and fluorobenzene networks both actually report slightly increased selectivity at higher pressures, though this is a result of the poor uptakes of both gases at these pressures.

**Figure 5:**
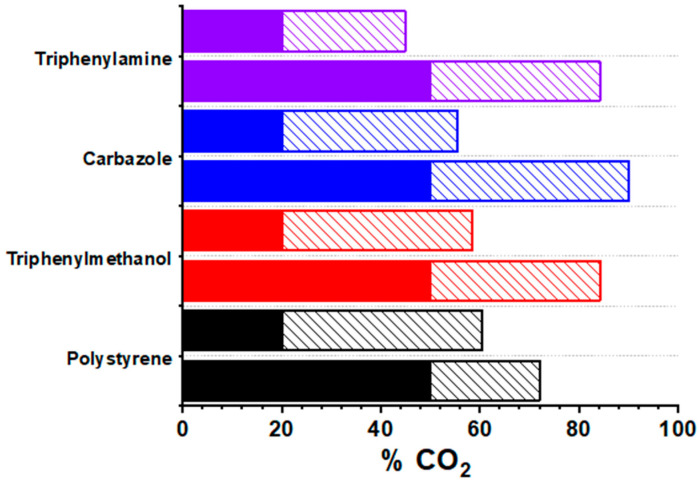
CO_2_ concentration of the input gas (solid bar) and the output exhaust gas stream at 1 bar (dashed bars) at 40 °C.

While some insight into the selectivity of the materials can be derived using pure gas streams, the use of mixed gas streams is more representative of actual industrial flue gas. To investigate how the materials performed at enriching a CO_2_ stream, the most promising materials were exposed to a gas mix composed of 80:20 N_2_:CO_2_ at 40 °C and 20 bar for 5 min. The concentration of CO_2_ in the output gas at 20 bar was measured using an IR sensor, after which the pressure was then released from the adsorber. When the pressure reached 1 bar, the concentration of CO_2_ was measured again. Finally, the same experiment was repeated using a stream composed of a 50:50 N_2_:CO_2_ mix at 40 °C (Figure 5). This test replicated two cycles, whereby the output from the first cycle is fed back into the PSA setup and the method is repeated once again.

Initially, when the chosen samples were exposed to an 80:20 N_2_:CO_2_ stream, all materials tested were able to selectively adsorb the CO_2_ at 20 bar and then desorb it at 1 bar. This resulted in the successful separation of CO_2_ from N_2_ and the generation of a gas stream enriched to over 50% CO_2_ in the case of triphenylmethanol, poly(styrene) and carbazole after one cycle. We therefore exposed the materials to a 50:50 N_2_:CO_2_ stream, the equivalent of feeding the stream from the first test back into the materials and repeated the experiment again. The triphenylmethanol and triphenylamine samples were able to enrich the stream of gas to over 80% CO_2_. This experiment demonstrates that these materials are able to take a dilute stream of flue gas and, after two pressure swing cycles, convert this dilute stream into a concentrated CO_2_ stream by preferential adsorption of CO_2_ over N_2_.

**Figure 6:**
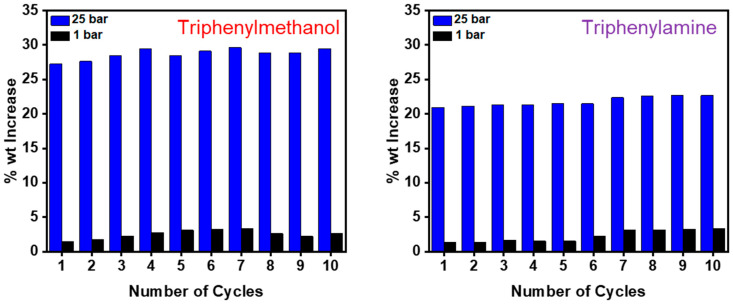
Recyclability studies for triphenylmethanol (left) and triphenylamine (right) networks during 10 adsorb/desorb cycles of CO_2_ at 25 bar and 40 °C.

Finally, the ability of the sorbents to be used over repeat adsorption–desorption cycles was tested using the best-performing triphenylmethanol and triphenylamine networks (Figure 6). These networks were exposed to a 25-bar stream of CO_2_ before having the pressure reduced to 1 bar with the uptakes at each pressure recorded and repeating for 10 cycles. Importantly, as in a typical PSA process, the materials were not exposed to a vacuum between runs to remove any CO_2_ so as not to further increase the energy demands of the process. Both materials reached a maximum uptake at 25 bar, and this was found to be reproducible over the 10-cycle run, demonstrating no loss in performance over time. Both samples retained some gas at 1 bar, though this quantity was minimal (<3% wt.) and had no significant effect on the uptake at higher pressures.

## 3. Conclusions

To conclude, a series of functional porous materials synthesized via conventional hypercrosslinking chemistry were applied as sorbents to selectively separate CO_2_ from simulated flue gas mixes. The uptake capacity, uptake rate and CO_2_:N_2_ selectivity at high pressure were all thoroughly examined to test the materials at high pressure and using a pressure swing approach. The best-performing materials were then taken forward and applied toward actual pressure swing separation experiments using simulated gas mixtures representative of those in industry. Finally, the recyclability of the optimum materials was tested to investigate if their performance was hindered after multiple adsorb/desorb cycles. All materials were found to uptake CO_2_ rapidly, with most of the uptake being complete within 2 min with the –OH functionalized and non-functional poly(styrene) network showing the highest CO_2_ capacity. Due to their high and selective uptakes, both the triphenylmethanol and triphenylamine networks were taken forward and applied to an actual pressure swing approach, where it was found that after only two cycles, they were able to convert a 20% CO_2_ stream into one exceeding 85% CO_2_. This was an excellent example of how cheaply synthesized porous materials can be easily synthesized and applied to a pressure swing methodology, demonstrating excellent CO_2_:N_2_ capabilities. It is hoped that this work inspires more research into PSA techniques to improve on the current energy-intensive and fiscally demanding temperature swing techniques rife throughout the industry.

## Figures and Tables

**Table 1:** Gas sorption properties of HCP networks.

Network	SA_BET_ (m^2^/g) ^a^	V_tot_ (cm^3^/g) ^b^	V_0.1_ (cm^3^/g) ^c^	V_0.1_/V_tot_
Poly(styrene)	1124	1.01	0.42	0.42
Triphenylmethanol	781	0.48	0.30	0.63
BINOL	888	0.45	0.35	0.70
Dibenzyl ether	397	0.39	0.14	0.36
Carbazole	445	0.24	0.17	0.71
Triphenylamine	630	0.37	0.24	0.65
Fluorobenzene	213	0.14	0.10	0.71

^a^ Apparent BET surface areas calculated at P/P_0_ = 0.01–0.15. ^b^ Total pore volume at 0.99 P/P_0_. ^c^ Micropore volume at 0.1 P/P_0._

**Table 2:** Average CO_2_ and N_2_ uptake of HCP networks at 40 °C.

Network	CO_2_ Uptake (wt.%)		N_2_ Uptake (wt.%)	
	10 bar	20 bar	10 bar	20 bar
Poly(styrene)	15.59	24.94	1.20	2.79
Triphenylmethanol	15.25	28.91	2.20	6.13
BINOL	10.16	18.64	5.92	8.87
Dibenzyl ether	6.84	10.82	0.93	1.31
Carbazole	17.44	22.41	2.48	4.41
Triphenylamine	13.75	21.04	1.45	2.98
Fluorobenzene	6.86	11.89	0.82	1.17

## Data Availability

The data presented in this study are available in the supplementary information.

## Supplementary Materials

The following are available online at https://www.mdpi.com/1996-1944/14/7/1605/s1, Figure S1: FTIR spectra, Figure S2: Solid state NMR, Figure S3: Nitrogen gas sorption isotherms, Figure S4: High pressure CO2 uptakes, Figure S5: High Pressure N2 uptakes, Figure S6 Apparatus setup Table S1: HCP prepation, Table S2: FTIR, Table S3: Sample masses, Table S4: Kinetic 10 Bar 25 °C CO2 uptakes, Table S5 Kinetic 20 bar 25 °C CO2 uptakes, Table S6: Kinetic 10 bar 40 °C CO2 uptakes, Table S7: Kinetic 20 bar 40 °C CO2 uptakes, Table S8: High pressure CO2 uptake at 25 °C, Table S9: High pressure CO2 uptake at 40 °C, Table S10: High pressure N2 uptake at 25 °C, Table S11: High pressure N2 uptake at 40 °C, Table S12: Pressure Swing, Table S13: Recyclability.
