# Supplementary material for: A Pressure Swing Approach to Selective CO2 Sequestration Using Functionalized Hypercrosslinked Polymers
Source: Materials (Basel). 2021 Mar 25;14(7):1605. doi: 10.3390/ma14071605 (PMC8036798; doi:10.3390/ma14071605)
Supplement: Supplementary file 1 [file materials-14-01605-s001.pdf]

materials

MDPI

Article

A Pressure Swing Approach to Selective CO_2 Sequestration Using Functionalized Hypercrosslinked Polymers

Alex M. James [1], Jake Reynolds [1], Daniel G. Reed [2], Peter Styring [2] and Robert Dawson [1,*]

[1] Department of Chemistry, University of Sheffield, Brook Hill, S3 7HF Sheffield, UK; alex.james@liverpool.ac.uk (A.M.J.); jreynolds5@sheffield.ac.uk (J.R.)
[2] Department of Chemical and Biological Engineering, University of Sheffield, Mappin Street, S1 3DJ Sheffield, UK; Cpa08dr@sheffield.ac.uk (D.G.R.); p.styring@sheffield.ac.uk (P.S.)
* Correspondence: r.dawson@sheffield.ac.uk; Tel.: +44-114-222-9357

Citation: James, A.M.; Reynolds, J.; Reed, D.G.; Styring, P.; Dawson, R. A Pressure Swing Approach to Selective CO_2 Sequestration Using Functionalized Hypercrosslinked Polymers. *Materials* **2021**, *14*, x. https://doi.org/10.3390/xxxxx

Academic Editor: Francesca Lionetto

Received: 18 February 2021
Accepted: 22 March 2021
Published: date

Publisher's Note: MDPI stays neutral with regard to jurisdictional claims in published maps and institutional affiliations.

Copyright: © 2021 by the authors. Licensee MDPI, Basel, Switzerland. This article is an open access article distributed under the terms and conditions of the Creative Commons Attribution (CC BY) license (http://creativecommons.org/licenses/by/4.0/).

Table S1. Preparation of FHCP networks.

Network	Monomer (g)	FDA (mL)	$FeCl_3$ (g)	DCE (mL)	% Yield
Triphenylmethanol	3.00	7.65	14.01	60	62
BINOL	2.89	5.31	9.73	50	92
Carbazole	3.34	7.10	12.98	50	107
Triphenylamine	3.68	7.96	14.60	60	55
Dibenzyl ether	3.00	6.70	12.28	50	68
Fluorobenzene	3.00	6.91	12.65	50	69
Poly(styrene)	3.46	5.90	11.23	50	110

FTIR Data

Presented in this section is all the FTIR data recorded for each of the polymer networks synthesised. All polymers display stretches at 1500–1700 cm^{-1} which can be ass igned to the C=C aromatic stretches of the polymer networks.[1] Likewise, peaks at ca. 2800 cm^{-1}, attributed to C–H alkyl stretches, and at 1480–1400 cm^{-1}, attributed to CH_2 bending vibrations, are present as a consequence of the newly introduced methylene bridges from the hypercrosslinking reaction. Peaks which are functional group dependant and therefore unique to networks are presented in table S2 along with the IR spectra for each network (Figure S1).

Table S2. Network dependant IR peaks found in each polymer network synthesised.

Functionality	Network	Wavenumber (cm^{-1})	Peak Label
-OH	Triphenylmethanol	3450	Alcohol OH stretch
	Triphenylmethanol	1200	Alcohol C-OH stretch
	BINOL	3450	Alcohol OH stretch
	BINOL	1200	Alcohol C-OH stretch
$-NR_x$	Carbazole	3350	N-H 2^0 amine stretch
	Carbazole	1340	C_{Ar}-N stretch
	Triphenylamine	3400	N-H 1^0 amine stretch
	Triphenylamine	1250	C_{Ar}-N stretch
R-O-R	Dibenzyl ether	1200	Alkyl aryl ether stretch
	Dibenzyl ether	1050	Alkyl aryl ether stretch
Ph-X	Fluorobenzene	1250	C_{Ar}-F stretch

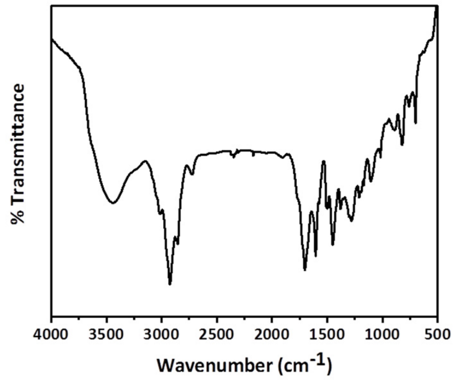

Figure S1a IR spectra of poly(styrene).

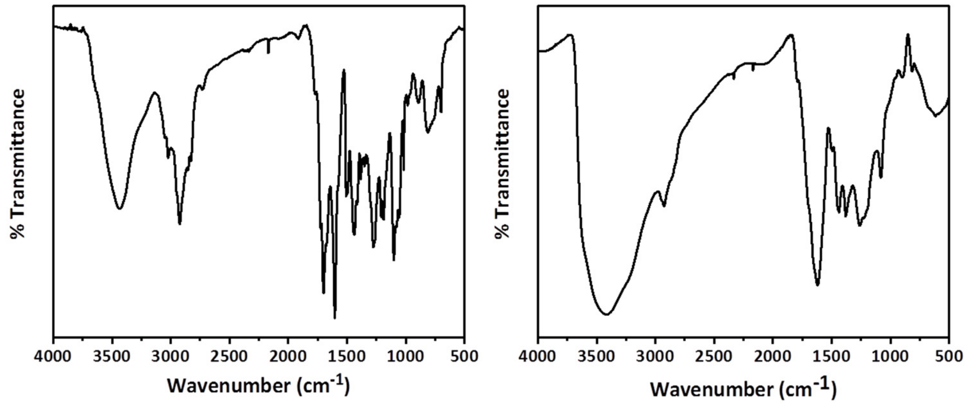

Figure S1b IR spectra of triphenylmethanol (left) and BINOL (right).

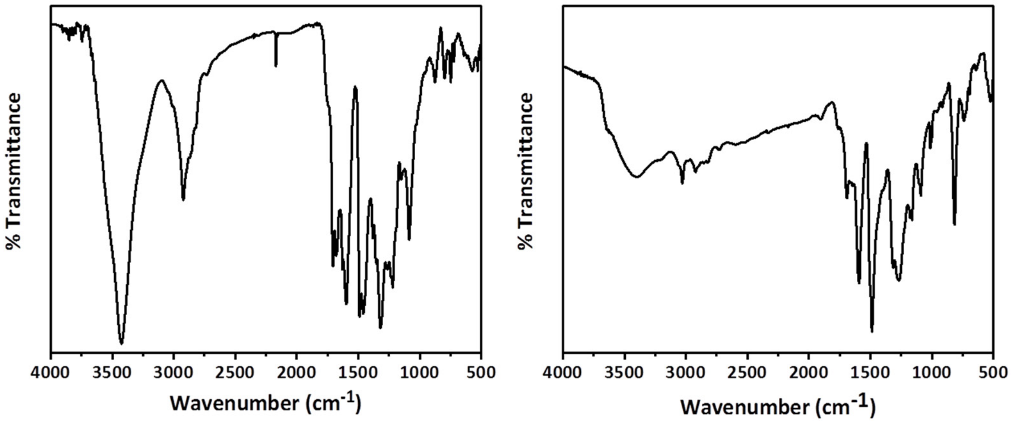

Figure S1c IR spectra of carbazole (left) and triphenylamine (right).

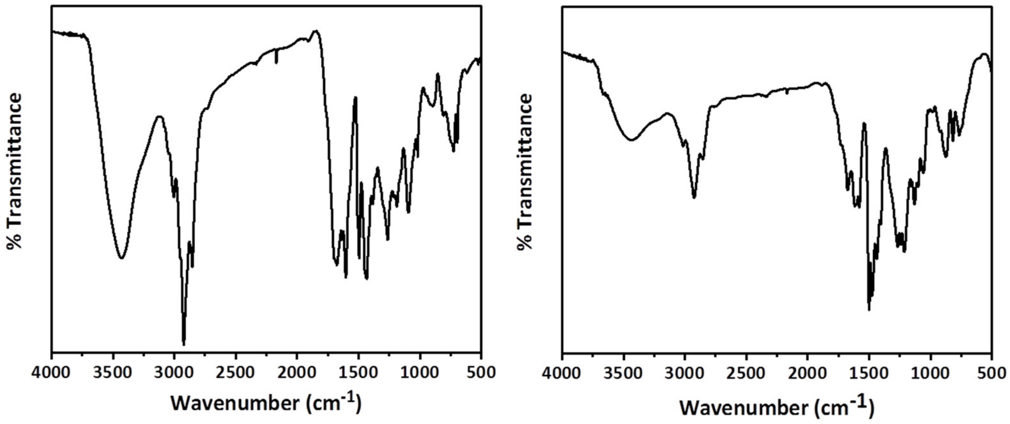

Figure S1.d IR spectra of dibenzyl ether (left) and fluorobenzene (right).

Solid-state NMR data

All polymer networks were analysed via ^{13}C–CP/MAS solid state NMR in order to further elucidate their structure and show monomer incoropration into the final networks. Peaks at low ppm (ca. 0–60) are present due to the –CH_2– groups from the external crosslinker. All other peaks are present due to the monomer and can be seen below:

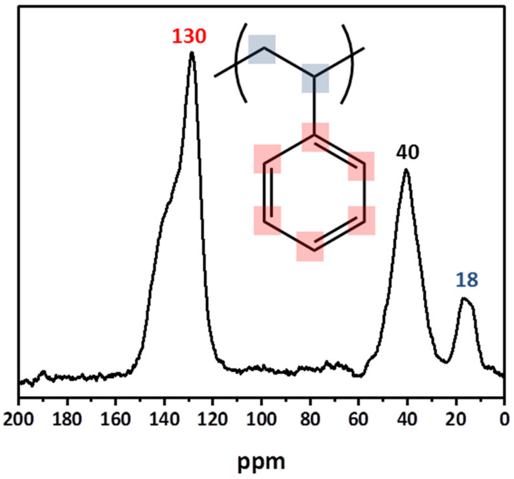

Figure S2a Solid-state NMR data spectra of poly(styrene).

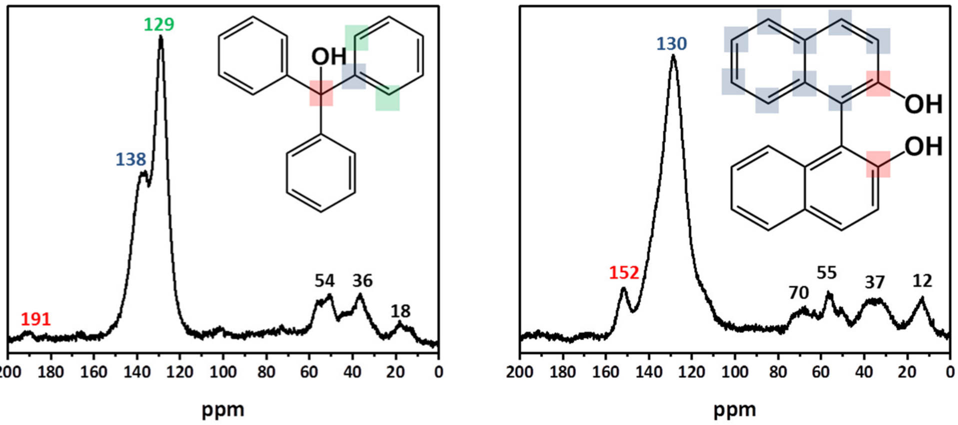

Figure S2b Solid-state NMR data spectra of triphenylmethanol (left) and BINOL (right).

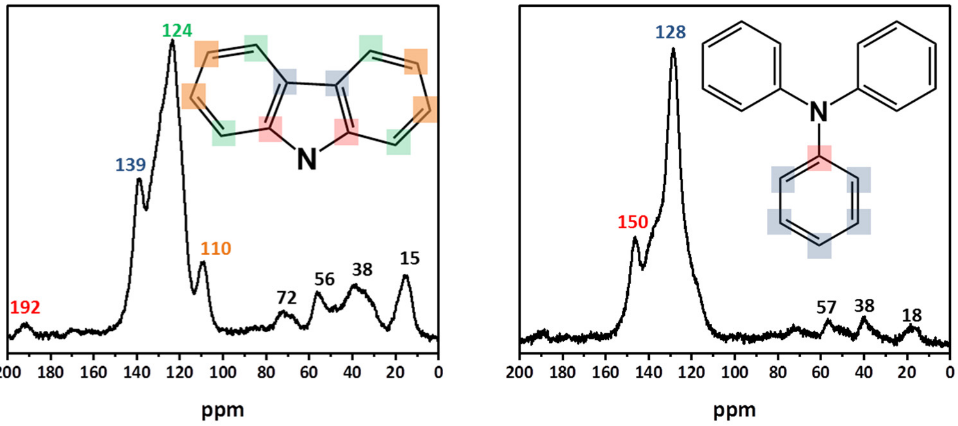

Figure S2c Solid-state NMR data spectra of carbazole (left) and triphenylamine (right).

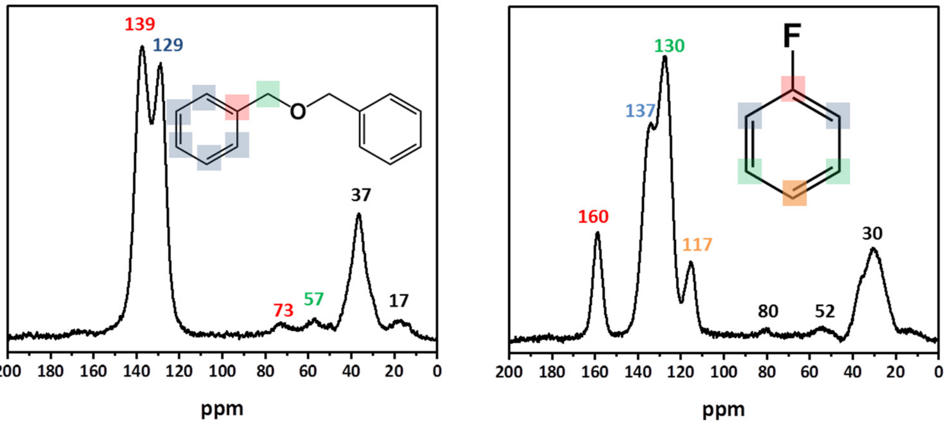

Figure S2d Solid-state NMR data spectra of dibenzyl ether (left) and fluorobenzene (right).

Gas sorption isotherms

The surface area of all polymer networks were determined through sorption of N_2 at 77 K and calculated over the pressure range of 0.01–0.15 P/P_0. Pore size distributions were also calculated for each network using NLDFT by applying the N_2 on carbon slit pores model. This data can be seen below for each network synthesised.

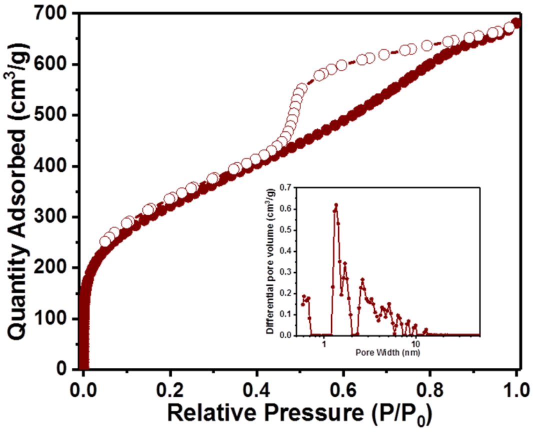

Figure S3a Full nitrogen isotherms of poly(styrene). Inset is the pore size distribution.

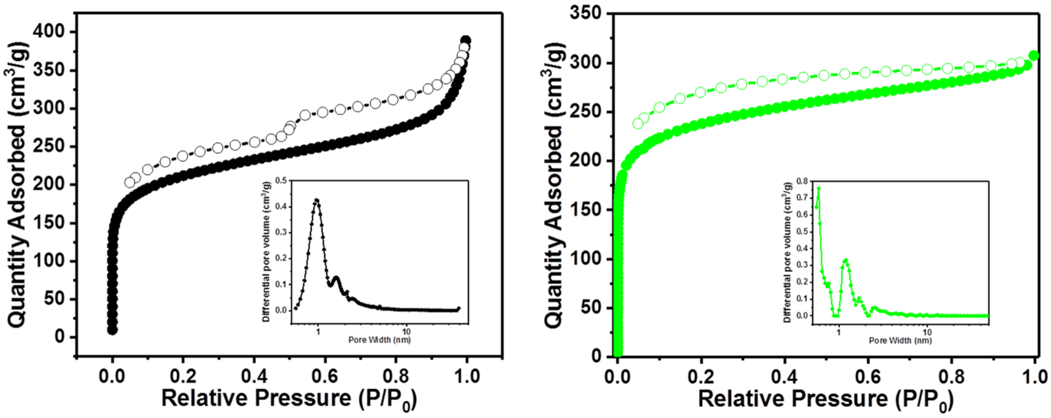

Figure S3b Full nitrogen isotherms of triphenylmethanol (left) and BINOL (right). Inset is the pore size distribution.

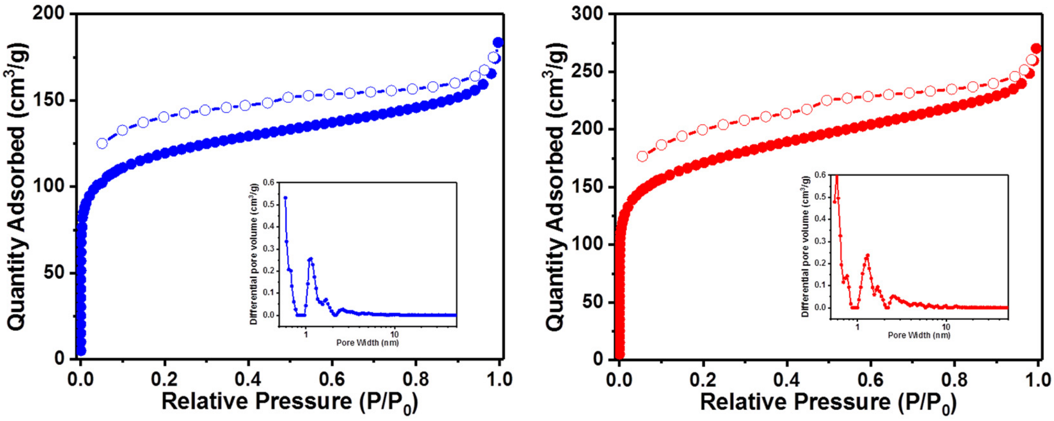

Figure S3c Full nitrogen isotherms of carbazole (left) and triphenylamine (right). Inset is the pore size distribution.

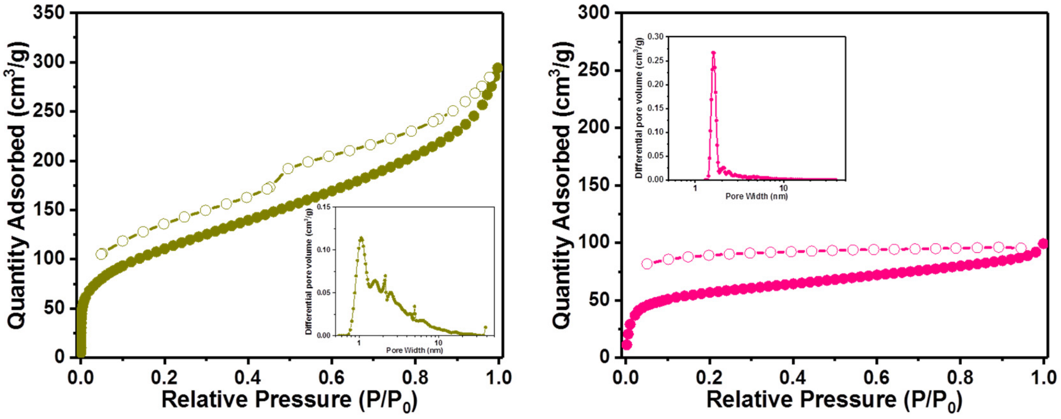

Figure S3d Full nitrogen isotherms of dibenzyl ether (left) and fluorobenzene (right). Inset is the pore size distribution.

Carbon Dioxide Uptake Data

All data obtained from experiments with CO_2 such as rate tests and pressure related uptake tests are presented in this section.

Table S3. The amount of sample used to determine the rate of uptake (kinetic tests) for each network.

Polymer Network.	Quantity (g)
Poly(styrene)	1.670
Triphenylmethanol	1.005
BINOL	0.631
Triphenylamine	1.799
Carbazole	1.011
Diethyl ether	2.797
Fluorobenzene	2.789

Kinetic test: 25 °C, 10 bar

Table S4a The results obtained through exposing poly(styrene) to 10 bar of CO_2 at 25 °C.

Time (s)	CO_2 Uptake (g)	% wt Uptake
0.00	0	0.000
10.35	0.432	19.840
20.36	0.486	23.070
30.15	0.495	23.610
60.55	0.53	25.710
120.45	0.568	27.990
301	0.607	30.320
600.56	0.638	32.170
900.44	0.64	32.290

Table S4b The results obtained through exposing triphenylmethanol to 10 bar of CO_2 at 25 °C.

Time (s)	CO_2 Uptake (g)	% wt Uptake
0.00	0.000	0.000
10.37	0.273	19.839
20.24	0.494	23.073
30.29	0.500	23.611
60.12	0.502	25.707

120.15	0.503	27.983
300.22	0.498	30.318
600.34	0.483	32.174
900.44	0.458	32.294

Table S4c The results obtained through exposing BINOL to 10 bar of CO_2 at 25 °C.

Time (s)	CO_2 Uptake (g)	% wt Uptake
0.00	0.000	0.000
10.26	0.410	17.145
20.04	0.408	39.135
30.16	0.406	39.732
60.6	0.404	39.931
120.01	0.446	40.031
300.13	0.443	39.533
600.34	0.446	38.041
900.09	0.440	35.553

Table S4d The results obtained through exposing carbazole to 10 bar of CO_2 at 25 °C.

Time (s)	CO_2 Uptake (g)	% wt Uptake
0.00	0.000	0.000
10.32	0.442	33.760
20.28	0.445	34.057
30.29	0.447	34.254
60.1	0.455	35.046
120	0.465	36.035
300.09	0.455	35.046
600.13	0.437	33.265
900.32	0.433	32.870

Table S4e The results obtained through exposing triphenylamine to 10 bar of CO_2 at 25 °C.

Time (s)	CO_2 Uptake (g)	% wt Uptake
0.00	0.000	0.000
10.48	0.354	14.081
20.1	0.42	17.749
30.47	0.451	19.473
60.09	0.458	19.862
120.2	0.47	20.529
300.09	0.47	20.529
600.06	0.494	21.863
900.12	0.495	21.918

Table S4f The results obtained through exposing dibenzyl ether to 10 bar of CO_2 at 25 °C.

Time (s)	CO_2 Uptake (g)	% wt Uptake
0.00	0.000	0.000
10.13	0.352	8.985
20.23	0.411	11.094
30.9	0.438	12.060
60.37	0.453	12.596
120.39	0.467	13.097

300.62	0.479	13.526
600.42	0.479	13.526
900.23	0.479	13.526

Table S4g The results obtained through exposing fluorobenzene to 10 bar of CO_2 at 25°C.

Time (s)	CO_2 Uptake (g)	% wt Uptake
0.00	0.000	0.000
10.23	0.340	8.581
20.14	0.424	11.592
30.3	0.454	12.668
60.15	0.478	13.529
120.52	0.502	14.389
300.31	0.522	15.106
600.87	0.53	15.393
900.56	0.538	15.680

Kinetic test: 25 °C, 20 bar

Table S5a The results obtained through exposing poly(styrene) to 20 bar of CO_2 at 25 °C.

Time (s)	CO_2 Uptake (g)	% wt Uptake
0.00	0.000	0.000
10.36	0.577	22.249
20.45	0.625	25.124
40.36	0.660	27.220
60.38	0.674	28.058
120.86	0.755	32.908
301.05	0.768	33.687
600.46	0.770	33.806
900.61	0.761	33.267

Table S5b The results obtained through exposing triphenylmethanol to 20 bar of CO_2 at 25 °C.

Time (s)	CO_2 Uptake (g)	% wt Uptake
0.00	0.000	0.000
10.36	0.458	40.026
20.13	0.509	48.109
30.12	0.514	48.901
60.28	0.517	49.377
120.36	0.52	49.852
300.41	0.532	51.754
600.27	0.54	53.022
900.35	0.542	53.339

Table S5c The results obtained through exposing BINOL to 20 bar of CO_2 at 25 °C.

Time (s)	CO_2 Uptake (g)	% wt Uptake
0.00	0.000	0.000
10.22	0.457	49.019
20.18	0.457	48.702
30.17	0.46	48.385
60.29	0.465	48.068
120.00	0.465	54.724

300.09	0.47	54.249
600.15	0.473	54.724
900.14	0.456	53.774

Table S5d The results obtained through exposing carbazole to 20 bar of CO_2 at 25 °C.

Time (s)	CO_2 Uptake (g)	% wt Uptake
0.00	0.00	0.000
10.37	0.641	43.083
20.09	0.644	43.379
30.09	0.644	43.379
60.04	0.651	44.072
120.07	0.659	44.863
300.11	0.647	43.676
600.03	0.645	43.478
900.15	0.648	43.775

Table S5e The results obtained through exposing triphenylamine to 20 bar of CO_2 at 25 °C.

Time (s)	CO_2 Uptake (g)	% wt Uptake
0.00	0.000	0.000
10.04	0.604	22.155
20.21	0.620	23.044
30.01	0.640	24.156
59.99	0.643	24.323
120.12	0.654	24.934
300.34	0.693	27.102
600.06	0.685	26.657
900.48	0.690	26.935

Table S5f The results obtained through exposing dibenzyl ether to 20 bar of CO_2 at 25 °C.

Time (s)	CO_2 Uptake (g)	% wt Uptake
0.00	0.000	0.000
10.32	0.557	12.569
20.24	0.601	14.143
30.56	0.604	14.250
60.35	0.609	14.429
120.35	0.63	15.179
300.32	0.659	16.216
600.48	0.678	16.895
900.68	0.673	16.717

Table S5g The results obtained through exposing fluorobenzene to 20 bar of CO_2 at 25 °C.

Time (s)	CO_2 Uptake (g)	% wt Uptake
0.00	0.000	0.000
10.13	0.553	12.462
20.41	0.683	17.123
30.71	0.708	18.020
60.21	0.731	18.844
120.4	0.772	20.314
300.34	0.807	21.569

600.11	0.818	21.964
900.78	0.844	22.896

Kinetic test: 40 °C, 10 bar

Table S6a The results obtained through exposing poly(styrene) to 10 bar of CO_2 at 40 °C.

Time (s)	CO_2 Uptake (g)	% wt Uptake
0.00	0.000	0.000
10.33	0.216	6.184
20.59	0.243	7.801
30.31	0.248	8.070
60.09	0.265	9.118
120.36	0.284	10.256
300.56	0.304	11.424
600.57	0.319	12.352
900.49	0.320	12.412

Table S6b The results obtained through exposing triphenymethanol to 10 bar of CO_2 at 40 °C.

Time (s)	CO_2 Uptake (g)	% wt Uptake
0.00	0.000	0.000
10.42	0.219	16.842
20.94	0.233	19.061
30.02	0.27	24.925
60.04	0.272	25.242
120.08	0.275	25.717
300.09	0.276	25.876
600.25	0.274	25.559
900.56	0.275	25.717

Table S6c The results obtained through exposing BINOL to 10 bar of CO_2 at 40 °C.

Time (s)	CO_2 Uptake (g)	% wt Uptake
0.00	0.000	0.000
10.42	0.183	11.137
20.56	0.183	11.137
30.04	0.200	13.831
60.65	0.200	13.831
120.74	0.213	15.891
300.31	0.216	16.367
600.3	0.215	16.208
900.75	0.218	16.684

Table S6d The results obtained through exposing carbazole to 10 bar of CO_2 at 40 °C.

Time (s)	CO_2 Uptake (g)	% wt Uptake
0.00	0.000	0.000
10.08	0.188	7.446
20.17	0.212	9.819
30.11	0.219	10.512
60.75	0.221	10.710
120.27	0.222	10.809
300.25	0.226	11.204

600.11	0.223	10.907
900.27	0.216	10.215

Table S6e The results obtained through exposing triphenylamine to 10 bar of CO_2 at 40 °C.

Time (s)	CO_2 Uptake (g)	% wt Uptake
0.00	0.000	0.000
10.1	0.256	7.964
20.31	0.311	11.021
30.1	0.332	12.189
60.49	0.353	13.356
120.18	0.375	14.579
300.07	0.390	15.413
600.17	0.391	15.468
900.01	0.390	15.413

Table S6f The results obtained through exposing dibenzyl ether to 10 bar of CO_2 at 40 °C.

Time (s)	CO_2 Uptake (g)	% wt Uptake
0.00	0.000	0.000
11.47	0.312	7.125
20.14	0.379	9.520
30.08	0.396	10.128
60.05	0.413	10.736
120.11	0.430	11.343
300.32	0.441	11.737
600.43	0.443	11.808
900.23	0.438	11.629

Table S6g The results obtained through exposing fluorobenzene to 10 bar of CO_2 at 40 °C.

Time (s)	CO_2 Uptake (g)	% wt Uptake
0.00	0.000	0.000
10.26	0.228	4.133
20.56	0.308	7.002
30.06	0.334	7.934
60.37	0.365	9.045
120.87	0.379	9.547
300.32	0.396	10.157
600.2	0.394	10.085
900.52	0.375	9.404

Kinetic test: 40 °C, 20 bar

Table S7a The results obtained through exposing poly(styrene) to 20 bar of CO_2 at 40 °C.

Time (s)	CO_2 Uptake (g)	% wt Uptake
0.00	0.000	0.000
10.33	0.592	22.009
20.34	0.598	22.368
30.38	0.602	22.608
60.29	0.620	23.686
120.73	0.645	25.183
300.94	0.670	26.680

600.96	0.676	27.039
900.21	0.680	27.279

Table S7b The results obtained through exposing triphenylmethanol to 20 bar of CO_2 at 40 °C.

Time (s)	CO_2 Uptake (g)	% wt Uptake
0.00	0.000	0.000
10.34	0.409	29.247
20.76	0.441	34.319
30.11	0.448	35.428
60.24	0.449	35.587
120.25	0.450	35.745
300.21	0.453	36.220
600.13	0.449	35.587
900.16	0.439	34.002

Table S7c The results obtained through exposing BINOL to 20 bar of CO_2 at 40 °C.

Time (s)	CO_2 Uptake (g)	% wt Uptake
0.00	0.000	0.000
10.29	0.324	15.777
20.29	0.335	17.520
30.34	0.332	17.045
60.32	0.340	18.312
120.74	0.354	20.531
300.61	0.354	20.531
600.45	0.360	21.482
900.12	0.361	21.640

Table S7d The results obtained through exposing carbazole to 20 bar of CO_2 at 40 °C.

Time (s)	CO_2 Uptake (g)	% wt Uptake
0.00	0.000	0.000
10.07	0.343	11.726
20.37	0.361	13.507
30.41	0.365	13.902
60.33	0.373	14.694
120.3	0.376	14.990
300.13	0.372	14.595
600.61	0.368	14.199
900.36	0.364	13.803

Table S7e The results obtained through exposing triphenylamine to 20 bar of CO_2 at 40 °C.

Time (s)	CO_2 Uptake (g)	% wt Uptake
0.00	0.000	0.000
10.28	0.547	17.929
20.26	0.549	18.041
30.1	0.586	20.097
60.00	0.586	20.097
120.89	0.610	21.431
300.06	0.628	22.432
600.07	0.628	22.432

900.1	0.630	22.543

Table S7f The results obtained through exposing dibenzyl ether to 20 bar of CO_2 at 40 °C.

Time (s)	CO_2 Uptake (g)	% wt Uptake
0.00	0.000	0.000
10.12	0.415	6.813
20.06	0.458	8.350
30.24	0.474	8.922
60.49	0.504	9.995
120.36	0.532	10.996
300.3	0.538	11.210
600.43	0.547	11.532
900.24	0.549	11.604

Table S7g The results obtained through exposing fluorobenzene to 20 bar of CO_2 at 40 °C.

Time (s)	CO_2 Uptake (g)	% wt Uptake
0.00	0.000	0.000
10.05	0.469	8.768
20.25	0.512	10.310
30.71	0.529	10.920
60.22	0.576	12.605
120.48	0.604	13.609
300.17	0.629	14.505
600.23	0.630	14.541
900.24	0.632	14.613

Table S7h The t_{90} values obatined for each polymer network at both 25 and 40 °C at 11 bar.

Material	T_{90} at 11 bar (s)	
	25 °C	40 °C
Triphenylmethanol	20	20
BINOL	10	10
Carbazole	10	10
Triphenylamine	30	20
Dibenzyl ether	60	120
Fluorobenzene	30	60

High pressure gas adsorption data

One the kinetic tests had been carried out and told us the times at which each networks were fully saturated with CO_2 the next step was to determine how varying pressures of gas affetced uptake. Each network was exposed to either CO_2 or N_2 at different pressures for a 5 minute adsorption period after which the material was weighted in order to determine sorption amounts. The data is presented both graphically and in tabular form in this section:

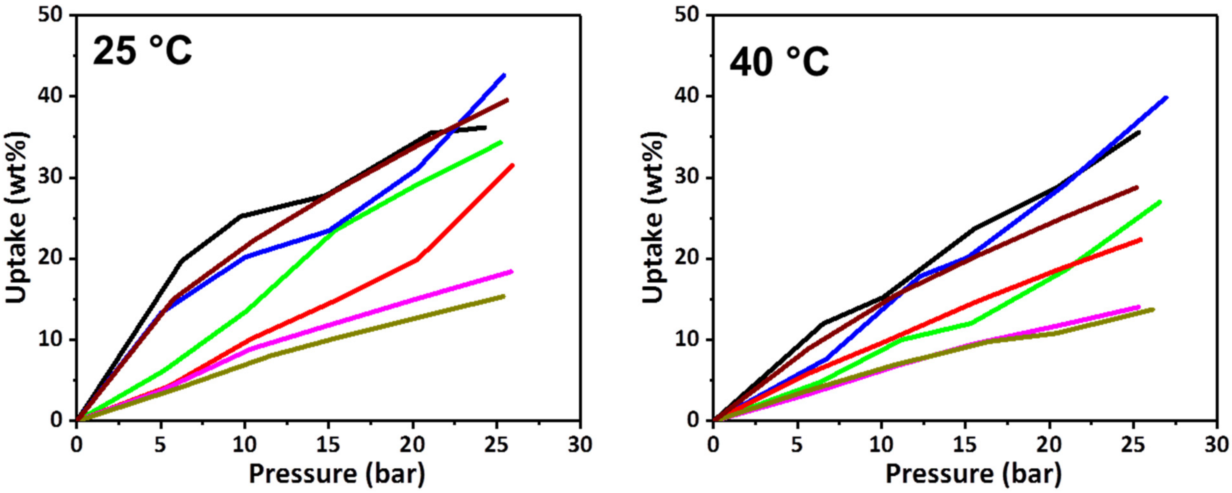

Figure S4. CO_2 uptakes at 25 and 40 °C for each network presented as a function of pressure. HCPs are coded as follows: poly(styrene), triphenylmethanol, BINOL, carbazole, triphenylamine, dibenzyl ether, and fluorobenzene.

CO_2 uptake at 25 °C

Table S8a CO_2 uptake of the poly(styrene) network as the pressure increases from 5 to 25 bar at 25 °C.

Pressure (bar).	% wt Uptake
0.000	0.000
5.750	15.036
10.530	22.250
15.430	28.411
20.690	34.404
25.610	39.547

Table S8b CO_2 uptake of the triphenylmethanol network as the pressure increases from 5 to 25 bar at 25 °C.

Pressure (bar)	% wt Uptake
0.000	0.000
6.240	19.711
9.770	25.231
14.820	27.808
21.080	35.477
24.280	36.163

Table S8c CO_2 uptake of the BINOL network as the pressure increases from 5 to 25 bar at 25 °C.

Pressure (bar)	% wt Uptake
0.000	0.000
5.170	6.196
10.100	13.562
15.400	23.529
20.220	29.088
25.210	34.300

Table S8d CO_2 uptake of the carbazole network as the pressure increases from 5 to 25 bar at 25 °C.

Pressure (bar)	% wt Uptake
0.000	0.000
5.050	13.384
10.020	20.209
15.010	23.445
20.300	31.114
25.430	42.604

Table S8e CO_2 uptake of the triphenylamine network as the pressure increases from 5 to 25 bar at 25 °C.

Pressure (bar)	% wt Uptake
0.000	0.000
5.360	3.297
10.460	14.970
15.250	21.625
20.370	25.935
25.500	31.836

Table S8f CO_2 uptake of the dibenzyl ether network as the pressure increases from 5 to 25 bar at 25 °C.

Pressure (bar)	% wt Uptake
0.000	0.000
6.020	4.051
11.540	8.055
15.500	10.262
20.170	12.677
25.400	15.364

Table S8g CO_2 uptake of the fluorobenzene network as the pressure increases from 5 to 25 bar at 25 °C.

Time (s)	% wt Uptake
0.000	0.000
5.010	3.740
10.350	8.867
15.130	11.905
20.260	15.079
25.860	18.420

CO_2 uptake at 40 °C

Table S9a CO_2 uptake of the poly(styrene) network as the pressure increases from 5 to 25 bar at 40 °C.

Pressure (bar).	% wt Uptake
0.000	0.000
5.680	8.963
10.890	15.585
15.690	20.360
20.690	24.943
25.190	28.782

Table S9b CO_2 uptake of the triphenylmethanol network as the pressure increases from 5 to 25 bar at 40 °C.

Pressure (bar)	% wt Uptake
0.000	0.000
6.530	12.009
10.100	15.251
15.600	23.782
20.530	28.911
25.310	35.588

Table S9c CO_2 uptake of the BINOL network as the pressure increases from 5 to 25 bar at 40 °C.

Pressure (bar)	% wt Uptake
0.000	0.000
5.170	4.838
10.100	10.164
15.400	12.311
20.220	18.643
25.210	26.924

Table S9d CO_2 uptake of the carbazole network as the pressure increases from 5 to 25 bar at 40 °C.

Pressure (bar)	% wt Uptake
0.000	0.000
6.700	9.959
10.300	17.436
15.140	19.882
20.830	22.409
26.940	28.354

Table S9e CO_2 uptake of the triphenylamine network as the pressure increases from 5 to 25 bar at 40 °C.

Pressure (bar)	% wt Uptake
0.000	0.000
5.350	8.932
10.300	13.745
15.550	17.399
20.500	21.042
25.850	25.162

Table S9f CO_2 uptake of the dibenzyl ether network as the pressure increases from 5 to 25 bar at 40 °C.

Pressure (bar)	% wt Uptake
0.000	0.000
5.470	3.671
10.620	6.839
16.360	9.796
20.430	10.817
26.150	13.753

Table S9g CO_2 uptake of the fluorobenzene network as the pressure increases from 5 to 25 bar at 40 °C.

Pressure (bar)	% wt Uptake
5.700	3.359
10.970	6.858
15.510	9.502
20.790	11.888
25.320	14.043
5.700	3.359

N_2 uptake at 25 °C

Table S10a N_2 uptake of the poly(styrene) network as the pressure increases from 5 to 25 bar at 25 °C.

Pressure (bar)	% wt Uptake
0.000	0.000
5.760	1.689
10.300	2.730
15.600	3.639
19.970	4.567
25.040	5.333

Table S10b N_2 uptake of the triphenylmethanol network as the pressure increases from 5 to 25 bar at 25 °C.

Pressure (bar)	% wt Uptake
0.000	0.000
5.740	0.117
10.060	0.257
15.120	0.375
20.010	1.022
25.750	1.995

Table S10c N_2 uptake of the BINOL network as the pressure increases from 5 to 25 bar at 25 °C.

Pressure (bar)	% wt Uptake
0.000	0.000
5.450	7.481
10.260	13.088
15.100	12.959
20.010	13.830
25.020	13.638

Table S10d N_2 uptake of the carbazole network as the pressure increases from 5 to 25 bar at 25 °C.

Pressure (bar)	% wt Uptake
0.000	0.000
5.710	0.512
10.700	3.545
15.500	5.035
20.200	7.137
25.200	8.593

Table S10e N_2 uptake of the triphenylamine network as the pressure increases from 5 to 25 bar at 25 °C.

Pressure (bar)	% wt Uptake
0.000	0.000
5.290	1.010
10.380	3.215
15.300	4.664
20.540	5.011
25.270	6.330

Table S10f N_2 uptake of the dibenzyl ether network as the pressure increases from 5 to 25 bar at 25 °C.

Pressure (bar)	% wt Uptake
0.000	0.000
5.500	2.545
10.370	2.604
15.860	2.678
20.990	2.902
25.940	2.999

Table S10g N_2 uptake of the fluorobenzene network as the pressure increases from 5 to 25 bar at 25 °C.

Pressure (bar)	% wt Uptake
0.000	0.000
5.310	0.795
10.230	1.213
15.750	1.287
20.250	1.296
25.710	1.466

N_2 uptake at 40 °C

Table S11a N_2 uptake of the poly(styrene) network as the pressure increases from 5 to 25 bar at 40 °C.

Time (seconds)	% wt Uptake
0.000	0.000
5.310	0.344
10.520	1.202
15.100	2.118
20.810	2.790
25.600	3.328

Table S11b N_2 uptake of the triphenylmethanol network as the pressure increases from 5 to 25 bar at 40 °C.

Pressure (bar)	% wt Uptake
0.000	0.000
5.740	1.765
10.050	2.196
15.580	5.311
20.860	6.129

25.030	7.719

Table S11c N_2 uptake of the BINOL network as the pressure increases from 5 to 25 bar at 40 °C.

Pressure (bar)	% wt Uptake
0.000	0.000
5.800	4.341
10.600	5.915
15.560	8.663
20.420	8.869
25.010	9.710

Table S11d N_2 uptake of the carbazole network as the pressure increases from 5 to 25 bar at 40 °C.

Pressure (bar)	% wt Uptake
0.000	0.000
5.680	1.854
10.530	2.480
15.080	3.400
20.950	4.411
25.340	6.783

Table S11e N_2 uptake of the triphenylamine network as the pressure increases from 5 to 25 bar at 40 °C.

Pressure (bar)	% wt Uptake
0.000	0.000
5.490	0.819
10.550	1.449
15.530	2.578
20.410	2.979
25.590	3.757

Table S11f N_2 uptake of the dibenzyl ether network as the pressure increases from 5 to 25 bar at 40 °C.

Pressure (bar)	% wt Uptake
0.000	0.000
5.500	0.026
10.680	0.932
15.950	1.193
20.470	1.308
25.230	1.451

Table S11g N_2 uptake of the fluorobenzene network as the pressure increases from 5 to 25 bar at 40 °C.

Pressure (bar)	% wt Uptake
0.000	0.000
5.660	0.672
10.820	0.827
15.960	1.089
20.270	1.169
25.260	1.204

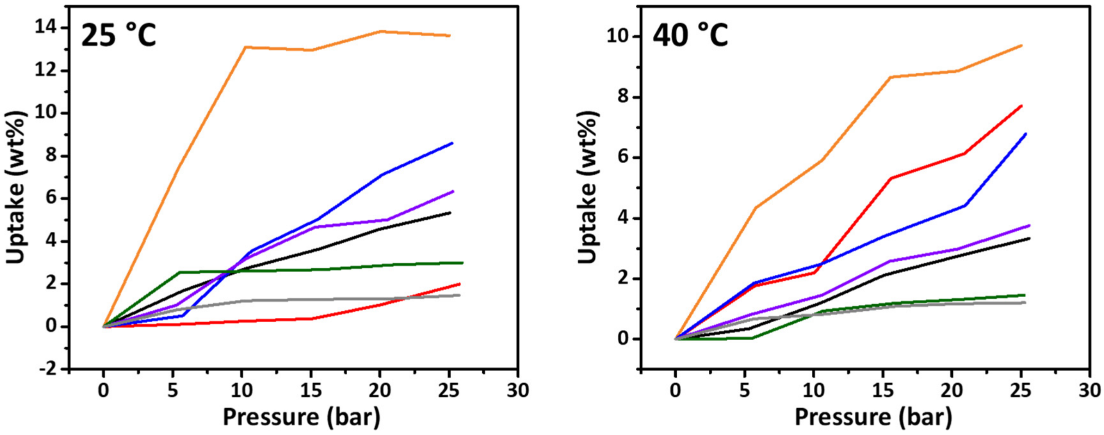

Figure S5. N_2 uptakes at 25 and 40 °C for each network presented as a function of pressure. HCPs are coded as follows: poly(styrene), triphenylmethanol, BINOL, carbazole, triphenylamine, dibenzyl ether, and fluorobenzene.

Pressure Swing Gas Adsorption Data

After testing of the materials with pure gases the next step was to examine the perfromance of select materials via a pressure swing methodology. This involved exposing the polymer to a mixture of CO_2:N_2 and then seeing how the material faired at separating the two gases out as the pressure is lowered from 20 bar to 1 bar. The data sems to indicate that the most promising materials, i.e. triphenylmethanol and carbazole seem to have better separation abilities at elevated temperatures. This is good when it comes to applying these materials to industrial processes given that 40 °C is a more realistic working temperature than 25 °C.

The raw data obtained from these experiments is shown below:

Table S12a Observed CO_2 concentrations after the polymers were exposed to a 20% CO_2 stream at 20 bar for 5 minutes before the pressure was dropped to 1 bar and the output stream analysed for CO_2 content at 25 °C.

**Polymer**	**% CO_2 Detected**	
	**20 bar**	**1 bar**
Triphenylmethanol	20.00	40.82
Carbazole	20.00	27.80
Triphenylamine	20.00	35.21
Dibenzyl ether	20.00	24.83
Fluorobenzene	20.00	32.13

Table S12b Observed CO_2 concentrations after the polymers were exposed to a 50% CO_2 stream at 20 bar for 5 minutes before the pressure was dropped to 1 bar and the output stream analysed for CO_2 content at 25 °C.

**Polymer**	**% CO_2 Detected**	
	**20 bar**	**1 bar**
Triphenylmethanol	50.00	80.04
Carbazole	50.00	63.53
Triphenylamine	50.00	93.90
Dibenzyl ether	50.00	99.68
Fluorobenzene	50.00	96.11

Table S12c Observed CO_2 concentrations after the polymers were exposed to a 20% CO_2 stream at 20 bar for 5 minutes before the pressure was dropped to 1 bar and the output stream analysed for CO_2 content at 40 °C.

Polymer	% CO_2 Detected	
	**20 bar**	**1 bar**
Triphenylmethanol	20.00	58.61
Carbazole	20.00	55.45
Triphenylamine	20.00	44.89
Dibenzyl ether	20.00	66.37
Fluorobenzene	20.00	46.96

Table S12d Observed CO_2 concentrations after the polymers were exposed to a 50% CO_2 stream at 20 bar for 5 minutes before the pressure was dropped to 1 bar and the output stream analysed for CO_2 content at 40 °C.

Polymer	% CO_2 Detected	
	**20 bar**	**1 bar**
Triphenylmethanol	50.00	84.25
Carbazole	50.00	89.97
Triphenylamine	50.00	84.20
Dibenzyl ether	50.00	99.06
Fluorobenzene	50.00	99.99

Recyclability

The final test carried out was a recyclability test of the sorbents so as to ensure that the materials still work without any noticeable loss of performance. To do this the polymer was exposd to a 20 bar stream of CO_2 for a 5 minutes adsorption period before being weighed. After this the gas was desorbed at 1 bar by simply opening the sorbent holder taps to let the CO_2 out. The material was again weighted to see if any CO_2 remianed present on the polymer. This was repeated 10 times and the results analyed so as to enure that the material still peforms well after 10 cycles. The data obtained from this experiment is reported below and shows no significant loss in performance by either material after 10 adsorb/desorb cycles with no treatment of the sorbent between runs:

Table S13a The data obtained from the recyclability test of triphenylmethanol.

Run	% wt CO_2 Adsorbed	
	**20 bar**	**1 bar**
1	43.956	10.047
2	45.997	10.989
3	47.096	10.832
4	46.311	11.146
5	47.410	11.617
6	47.881	11.146
7	48.352	11.460
8	47.253	11.303
9	49.137	10.675
10	49.922	11.303

Table S13b The data obtained from the recyclability test of carbazole.

Run	% wt CO_2 Adsorbed	
	**20 bar**	**1 bar**
1	46.010	5.443
2	47.116	5.953
3	47.711	5.868
4	47.286	6.038
5	47.881	6.293
6	48.136	6.038
7	48.391	6.208
8	47.796	6.123
9	48.816	5.783
10	49.242	6.123

Experimental

Materials

Anhydrous 1,2-dichloroethane (DCE, > 99%), iron (III) chloride (FeCl3, 97%) and formaldehyde dimethyl acetal (FDA, >99%), BINOL (>99%), dibenzyl ether (>99%) and poly(styrene) (Mn=280,000 g/mol) were all purchased from Sigma-Aldrich. Triphenylmethanol (Lancaster synthesis, >99%), carbazole (Alfa Aesar, 95%) and triphenylamine (Fluorochem >99%) were used as received. All chemicals were used as received unless stated otherwise.

Synthesis of HCPs

Hypercrosslinked porous polymers were synthesised via the "knitting route" using functional aromatic monomers. All reactions were performed under a nitrogen atmosphere (see Table S1 for details). Using triphenylmethanol as an example; triphenylmethanol (3.00 g, 11.54 mmol, 1 eq.) was added to a 2-necked round bottom flask which was degassed by three freeze-pump-thaw cycles. To this vessel DCE (60 mL) and FDA (7.65 mL, 86.57 mmol, 7.5 eq.) were added along with a slurry of FeCl3 (14.02 g, 86.57 mmol, 7.5 eq.) in DCE. The reaction was heated to 80 °C and left for 16 h to afford a solid black product. The crude black product was washed and filtered with methanol before being solvent extracted with methanol using Soxhlet apparatus overnight. The black solid was washed and filtered with chloroform and methanol before being left to dry overnight under vacuum at 60 °C.

Characterisation

Fourier transform infrared (FTIR) spectroscopy was performed using a Perkin-Elmer Spectrum 100 fitted with an attenuated total reflectance tip (ATR). Solid-State NMR samples were packed into 4 mm zirconia rotors and transferred to a Bruker Avance III HD spectrometer. 1D ^{1}H–^{13}C cross-polarisation magic angle spinning (CP/MAS) NMR experiments were measured at 125.76 MHz (500.13 MHz ^{1}H) at a MAS rate of 10.0 kHz. The 1H $\pi/2$ pulse was 3.4 µs, and two-pulse phase modulation (TPPM) decoupling was used during the acquisition. The Hartmann-Hahn condition was set using hexamethylbenzene. The spectra were measured using a contact time of 2.0 ms. The relaxation delay D1 for each sample was individually determined from the proton T1 measurement (D1 = 5 × T1). Samples were collected until sufficient signal to noise was observed, typically greater than 256 scans. The values of the chemical shifts are referred to that of TMS.

Gas sorption measurements to determine porosity and specific surface area were performed using a Micromeritics ASAP 2020 Plus analyser employing high purity gases. Approximately 100 mg of sample was degassed at 120 °C for 16 h under dynamic vacuum

immediately prior to analysis. BET surface areas were calculated using nitrogen gas at 77 K over a pressure range of 0.01–0.15 P/P_0.

High pressure adsorption experiments to determine N_2 and CO_2 uptake were carried out in an identical way to that previously reported by Reed et al.[70] using a bespoke packed-bed adsorption column constructed from Swagelok™ (Fig. 8) piping and fitting using a Jasco BP-1580-81 back pressure regulator, an Omega PX409USB High Accuracy Pressure Transducer, a 42AAV48 Midwest Pressure Systems Gas Pressure Booster, and an AND GF-1000 High Capacity 3 decimal place balance. In a typical experiment the packed adsorber bed was exposed to a specific pressure of high purity gas for a defined period of time by opening the inlet valve to introduce the gas stream. During this process, the outlet valve remined closed to ensure constant pressure hence the method was a static adsorption process and not a flow process. After the defined time period the valve was closed to the gas stream. The pressurised packed bed was isolated from the system and taken to the balance to determine the N_2/CO_2 uptake gravimetrically. To ensure only the actual uptake due to adsorption was measured the free space within this packed bed, now occupied by the gas the sample was exposed to was calculated and subtracted from the total mass to accurately determine the mass increase due to adsorption.

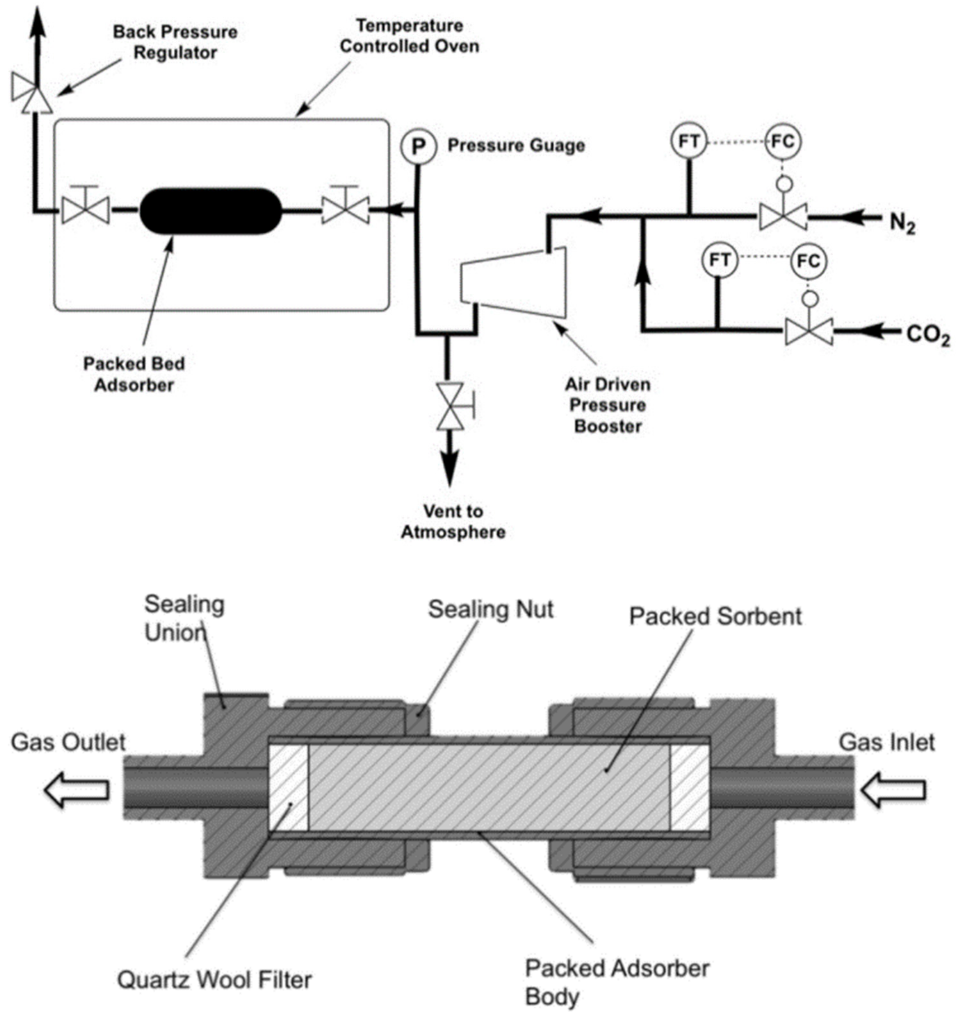

Figure S6. (Top) Simplified flow diagram of the experimental apparatus setup used during the high pressure testing. (Bottom) Cross-sectional view of the packed-bed adsorber used for CO_2 separation at high pressures. Figures reproduced with permission from ref.[70]. Copyright 2017, Reed, Dowson and Styring.

Given that uptake was determined gravimetrically it was important to calculate the weight of gas present which was not interacting with the sorbent. This is known as the void space. The void space was calculated before the experiments took place. The accurate internal volume of the adsorber (empty) was found by water displacement (VA). The adsorber rig was then weighed (empty) and under vacuum. Quartz wool was used to ensure that packed polymers were not ejected from the adsorber, and the accurate amount of quartz wool used was weighed and included in the empty mass of the adsorber bed. A portion of quartz wool was packed into one end of the adsorber and the polymer to be tested was then packed on top. The second portion of quartz wool was then added at the other end to seal the polymer in place and the adsorber was closed and sealed. The adsorber was then re-weighed to give the packed sorbent weight. The volumes of the sorbent (VS) and quartz wool (VQ) were obtained from pycnometer measurements. These volumes were subtracted from the total internal volume to give the void space as shown in Equation (1).

$$Void\ Space = V_A - (V_S + V_Q) \tag{1}$$

The CO_2 capacity of the sorbent was calculated using a static gas pressure and was carried out using pure CO_2 gas. The starting weight of the packed adsorber was taken before the gas was introduced. Pure CO_2 then enters the adsorber and the total weight increase of the system was determined (MT). This was achieved by closing the valves to the sorbent holder, removing it from the system and placing it on the balance, the mass of the empty assembly having previously been measured. The mass increase was attributed to the CO_2 that had been adsorbed onto the sorbent (M_{ads}) and CO_2 in the void space (M_{void}). In order to find the mass of CO_2 in the void space, the density of the gas at that specific pressure and temperature was determined. This void space mass (M_{void}) was removed from the total mass increase (M_T). The remaining mass (M_{ads}) was then attributed to the gas that had adsorbed onto the sorbent (Equation (2)).

$$M_{ads} = M_T - M_{Void} \tag{2}$$

For experiments involving higher temperatures, the exact process was repeated however the oven was set to the designated temperature and was left overnight to ensure the temperature was achieved and the sorbent solder was also at the set temperature. A thermometer was placed inside of the oven and was used to determine the temperature accurately in tandem with the thermostat build into the oven.

Live IR tracking was carried out via non-dispersive infrared absorption using a CM-40401 SprintIR6S high speed CO_2 sensor, capable of taking 20 readings per second accurate to 70 ppm, purchased from CO_2Meter. The detector was calibrated using a pure stream of N_2 gas. Data was analysed using GasLab® version 2.0.8.14 which allowed for CO_2 output to be presented as a % concentration.
